# Therapeutic potential of stem cells and acitretin on inflammatory signaling pathway-associated genes regulated by miRNAs 146a and 155 in AD-like rats

**DOI:** 10.1038/s41598-023-36772-3

**Published:** 2023-06-13

**Authors:** Emad M. Elzayat, Sherif A. Shahien, Ahmed A. El-Sherif, Mohamed Hosney

**Affiliations:** 1 Zoology Department, Faculty of Science, Cairo University, Giza, 12613 Egypt; 2 Biotechnology/Bimolecular Chemistry Program, Faculty of Science, Helwan University, Cairo, Egypt; 3 Department of Chemistry, Faculty of Science, Cairo University, Giza, 12613 Egypt

**Keywords:** Biotechnology, Molecular biology, Neuroscience, Zoology, Biomarkers, Diseases, Molecular medicine, Neurology

## Abstract

Alzheimer’s disease (AD) is one of the most common causes of dementia. Several drugs are used to improve the symptoms, but do not stop AD progression. There are more promising treatments that may have a significant role in AD diagnosis and treatment such as miRNAs and stem cells. The present study aims to develop a new approach for AD treatment by mesenchymal stem cells (MSCs) and/or acitretin with special reference to inflammatory signaling pathway as NF-kB and its regulator miRNAs in AD-like rat model. Fourty-five male albino rats were allotted for the present study. The experimental periods were divided into induction, withdrawal, and therapeutic phases. Expression levels of miR-146a, miR-155, necrotic, growth and inflammatory genes were assessed using RT-qPCR. Histopathological examination of brain tissues was performed in different rat groups. The normal physiological, molecular, and histopathological levels were restored after treatment with MSCs and/or acitretin. The present study demonstrates that the miR-146a and miR-155 might be used as promising biomarkers for AD. MSCs and/or acitretin proved their therapeutic potential in restoring the expression levels of targeted miRNAs and their related genes concerning NF-kB signaling pathway.

## Introduction

Dementia is a group of symptoms characterized by its contribution in the impairment of cognitive functions such as memory loss, changes in personality and behavior and learning difficulties accompanied by physical, social, and psychological burdens to the patients and their families. Currently, more than 55 million people suffer from dementia worldwide and this number increases nearly by 10 million new cases per year^1^. Dementia is considered as the seventh cause of death among all diseases and one of the main reasons of disability among older people^1^.

Alzheimer’s disease (AD) is the most common form of dementia and accounts for 60–70% of its cases^1^. Many studies reported that some risk factors, such as aging, family history, depression, head injury, oxidative stress, neuro-inflammation, environmental metals exposure, and cognitive activity, are associated with AD^2^.

Aluminum is one of the most common risk factors in which prolonged exposure to its toxicity and its accumulation in the brain may raise oxidative stress resulting in AD onset and neurons damage^3^. Despite the incomplete understanding of AD causes till now, two factors are assumed to play critical roles at the AD onset: amyloid beta plaques (Aβ) and neurofibrillary tangles (NFT)^4^. In healthy neurons, alpha- and gamma-secretase enzymes play an important role in the amyloid precursor protein (APP) digestion but when the β-secretase (BACE 1) combine with the gamma-secretase, this reaction produces an insoluble amyloid-beta peptides which clump together forming amyloid-beta plaques (AβP). Aβ plays a critical role in neuronal function and neurotoxicity, so, AβP accumulation in the brain causes cell damage and perturbs signaling process between neurons leading to deterioration in the brain activities and memory loss^5,6^.

Tau proteins are found on the microtubules which form neurons cytoskeleton. When tau proteins are phosphorylated, they undergo some conformational changes, leave the microtubules and clump together forming neurofibrillary tangles which accumulate in the brain of AD patients leading to neuronal loss. Consequently, the reduction of tau proteins in microtubule structure will result in microtubules weakness and disability in performing their signaling function leading eventually to apoptosis or cell death^7,8^.

Unfortunately, the clinical trials for the treatment of AD are not enough and their results are not fully satisfactory. Few classes of drugs are approved till now for AD treatment including acetylcholinesterase enzyme inhibitors and N-methyl d-aspartate (NMDA) antagonists. Acetylcholinesterase enzyme inhibitors are used for acetylcholine (ACh) degradation inhibition in the synapses by blocking cholinesterase enzymes, leading to accumulation of ACh and activation of cholinergic receptors^9–11^. NMDARs stimulation leads to Ca^2+^ influx which results in synaptic dysfunction, neuronal cell death and cognitive function impairments. Several NMDAR antagonists have been launched and tried clinically but unfortunately, most of them did not achieve the expected success due to lack of efficiency and side effects^12–14^. Most of these drugs for AD succeed to treat the symptoms, but do not stop AD progression.

On the other hand, there are more promising treatments that may have a significant role in AD diagnosis and treatment such as miRNAs and stem cells. microRNAs (MiRNAs) are small non-coding RNAs (21–25 nucleotides) that play a critical role in the post-transcriptional regulation of gene expression through post-transcriptional gene silencing and binding to 3′ and 5′ untranslated region (UTR) of the messenger RNAs (mRNAs) to degrade mRNA or block its translation^15^. Many studies reported that miRNAs in blood, CSF and brain are affected through upregulation or downregulation of their expression in AD patients and also influence some signaling pathways involved in AD progression by targeting APP or BACE 1 expression as well as their roles in neuronal function and synaptic transmission making them ideal candidates to be used as biomarkers for the diagnosis of AD at early stages and a new approach for AD treatment^16,17^. From the other hand, it has been reported that MSCs play a significant role in microglia modifications leading to an increment in the levels of anti-inflammatory cytokines such as IL-4 and IL-10, and decrement in the levels of pro-inflammatory cytokines such as IL-1β and TNF-α. Also, MSCs can degrade Aβ deposits in AD-like model that led to improve memory and cognitive functions making them a possible new approach for AD treatment^18^.

Acitretin, the primary FDA approved drug for Psoriasis vulgaris, is a candidate drug for AD treatment. It exhibits a considerable impact on some AD hallmarks as it upregulates α-secretase expression and increases activity in human neuronal cells and in mouse models of the disease altering amyloid-β (Aβ) peptides and plaques formation leading to improved behaviour and cognitive function. These observations led to the idea of using retinoids as therapeutic drugs in human AD patients^19^. Thereby, the present study aims to expose the therapeutic effect of mesenchymal stem cells (MSCs) and/or acitretin on NF-kB inflammatory signaling pathway-associated genes (TNFAIP1, TGF-βRII, IL-1β and IL-4) and its regulatory miRNAs namely, miR-146a and miR-155 in AD-like rat model. The novelty of the current investigation is finding one or more specific new biomarkers that could help for diagnostic, prognostic, and therapeutic purposes of Alzheimer’s disease. The present study hypothesizes that the AlCl_3_ accumulation provokes inflammation within the brain that ultimately affects some inflammatory signal pathways that end with neurodegeneration which is a major sign of AD. By evaluation the expression levels of some inflammatory cytokines and their regulatory miRNAs, one or more candidates can be found that help as bio-diagnostic and therapeutic markers for Alzheimer’s disease.

## Results

### Measurement of serum and brain Aβ1-42 levels

**Figure 1:**
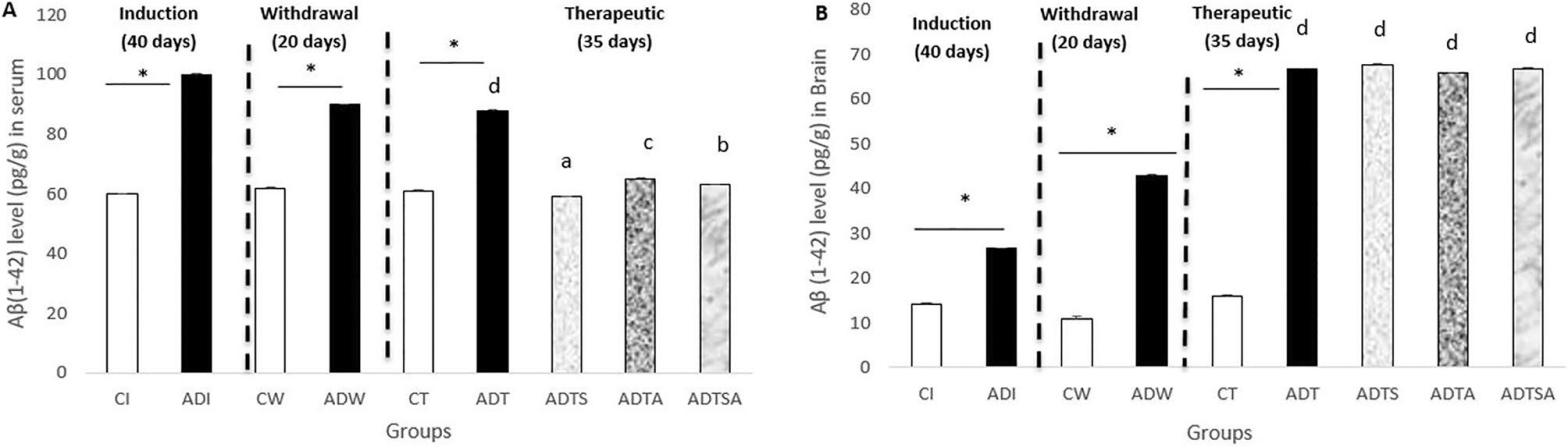
Variations in (**A**) serum beta-amyloid concentrations, (**B**) brain beta-amyloid concentrations in AD-like rat model throughout the whole experimental period, the expression of values is using mean ± SD; *: *P* ≤ 0.05 significant difference compared to both control and treated groups. One-way ANOVA followed by Duncan a, b post hoc Test was done, and statistical significance is expressed by different letters. Groups having different small letters are significantly different at *P* ≤ 0.05, while groups having the same small letters are non-significantly different *P* > 0.05.

Alzheimer’s disease induction (ADI) and Alzheimer’s disease withdrawal (ADW) rats showed a significant increment in serum Aβ1-42 levels compared to the respective control induction (CI) and control withdrawal (CW) rats at the end of both induction and withdrawal phases. Similarly, at the end of therapeutic phase, Alzheimer’s disease therapeutic (ADT) rats exhibited a significant increase in serum Aβ1-42 levels in comparison with the respective control therapeutic (CT) rats. Regarding the effects of MSCs and acitretin, Alzheimer’s disease therapeutic stem cells (ADTS), Alzheimer’s disease therapeutic acitretin (ADTA) and Alzheimer’s disease therapeutic stem cells and acitretin (ADTSA) rats showed nearly equal values of Aβ1-42 levels to control rats (CT) (*P* ≤ 0.05) (Fig. 1A).

On the other side, at the end of both induction and withdrawal phases, ADI and ADW rats exhibited a significant increment in brain Aβ1-42 levels in comparison with the respective control rats (CI and CW). Likewise at the end of therapeutic phase, untreated Alzheimer’s disease therapeutic group (ADT) showed the same trend of significant increase in Aβ1-42 levels against the respective control and was continuation to the induction and withdrawal phases (*P* ≤ 0.05). Concerning the effects of MSCs and acitretin, ADTS, ADTA and ADTSA rats showed no significant change in brain Aβ1-42 levels (*P* > 0.05) (Fig. 1B).

### Measurement of serum oxidative stress biomarkers

**Figure 2:**
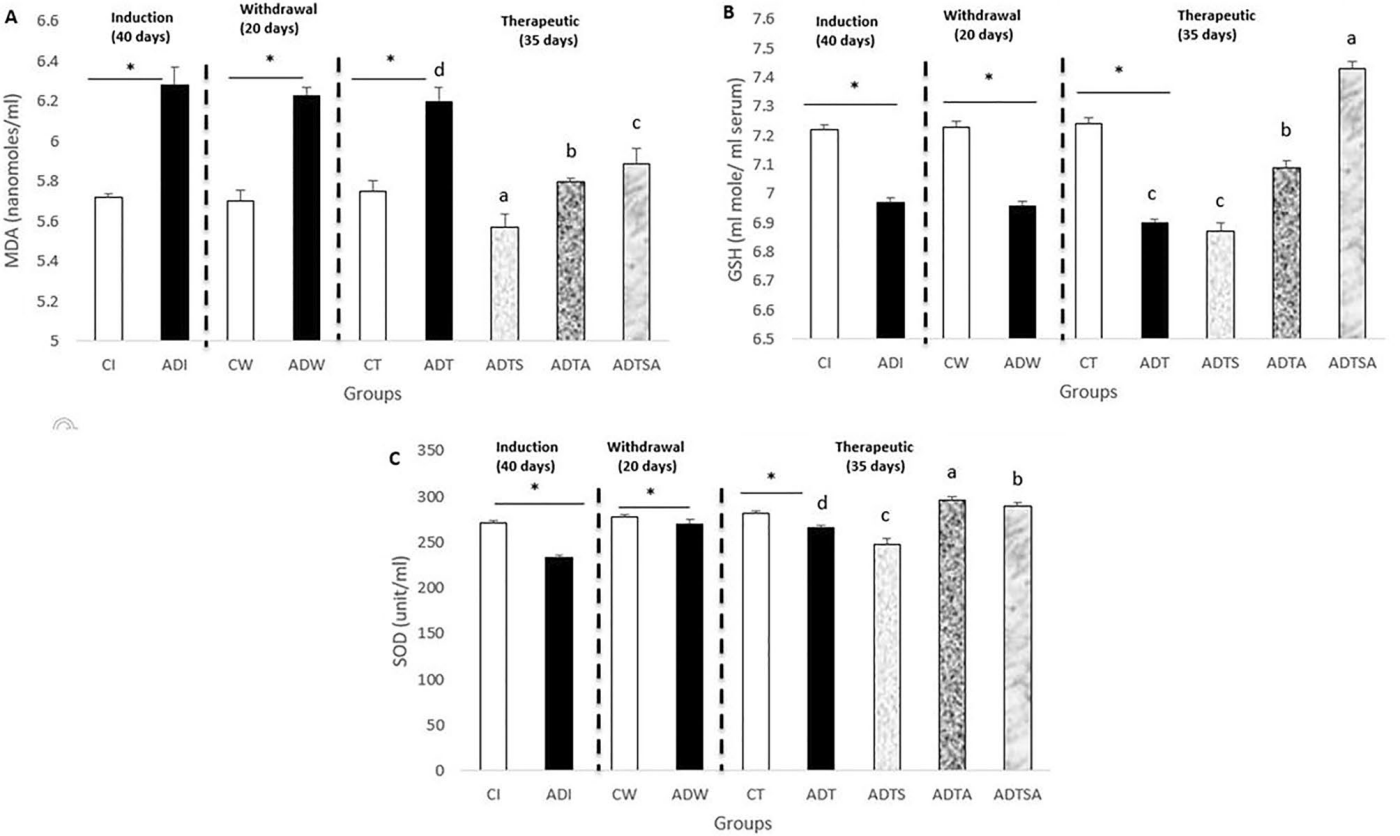
Variations in serum (**A**) MDA (**B**) GSH and (**C**) SOD in AD-like rat model throughout the whole experimental period, the expression of values is using mean ± SD; *: *P* ≤ 0.05 significant difference compared to both control and treated groups. One-way ANOVA followed by Duncan a, b post hoc Test was done, and statistical significance is expressed by different letters. Groups having different small letters are significantly different at *P* ≤ 0.05, while groups having the same small letters are non-significantly different *P* > 0.05.

At the end of both induction and withdrawal phases, ADI and ADW rats exhibited a significant increment in MDA levels in comparison with the respective control rats (CI and CW). In addition, at the end of therapeutic phase, untreated group (ADT) showed the same trend of significant increase in MDA levels compared to the respective control (CT) and was continuation to the induction and withdrawal phases. Regarding the effects of MSCs and acitretin, ADTS, ADTA and ADTSA rats significantly showed reduction in MDA levels against respective ADT group, and ADTS showed the best effect (*P* ≤ 0.05) (Fig. 2A).

As concerned to the antioxidant defense system as represented by GSH and SOD, ADI and ADW rats showed a significant decrease in GSH levels compared to the respective control rats (CT, CW) at the end of both induction and withdrawal phases. Similarly, ADT exhibited a considerable reduction in GSH levels in comparison with the respective control rats (CT). Concerning the effects of MSCs and acitretin, ADTA and ADTSA rats exhibited increase in GSH levels compared to ADT group, and ADTSA showed the best one (*P* ≤ 0.05) (Fig. 2B).

At the end of the induction phase, ADI exhibited a significant decrease in SOD levels compared to the respective controls (CI) (*P* ≤ 0.05), while ADW exhibited a non-significant change compared to the respective controls (CW) at the end of withdrawal phase. At the end of the therapeutic phase, untreated group (ADT) showed same trend of non-significant change in SOD levels against the respective control group (CT) (*P* > 0.05). Regarding the effects of MSCs and acitretin, ADTA and ADTSA rats showed a significant increment in SOD levels compared to ADT group, and ADTA showed the best effect (*P* ≤ 0.05) (Fig. 2C).

### Expression levels of miR-146a, miR-155, necrotic, growth and inflammatory genes in brain rat group

**Figure 3:**
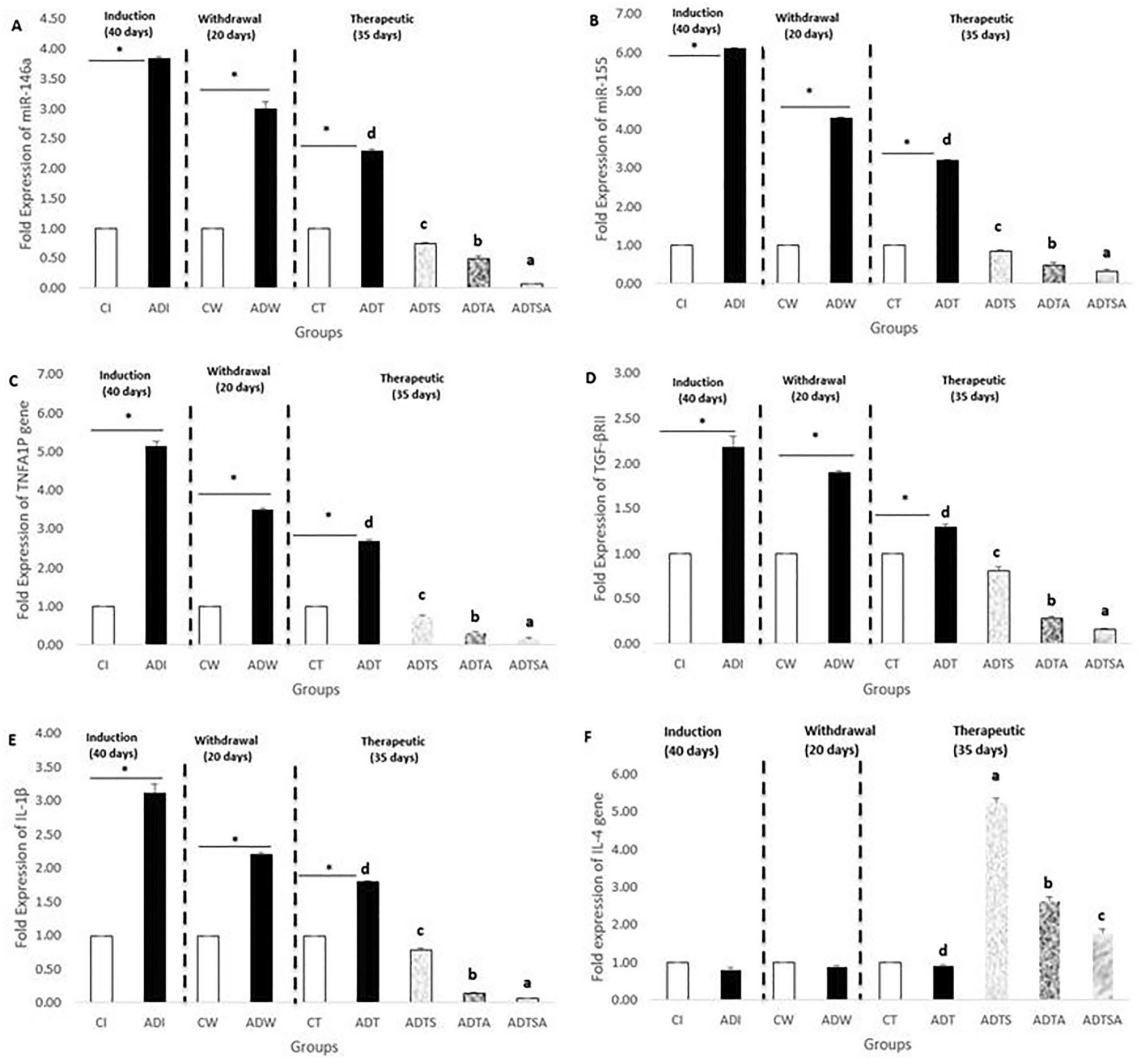
Fold expressions of brain (**A**) miR-146a (**B**) miR-155, (**C**) TNFAIP1, (**D**) TGF-βRII, (**E**) IL-1β, and (**F**) IL-4 in AD-like rat model throughout the whole experimental period, the expression of values is using mean ± SD; *P* ≤ 0.05 significant difference compared to both control and treated groups. *: *P* ≤ 0.05 significant difference compared to both control and treated groups. One-way ANOVA followed by Duncan a, b post hoc Test was done, and statistical significance is expressed by different letters. Groups having different small letters are significantly different at *P* ≤ 0.05, while groups having the same small letters are non-significantly different *P* > 0.05.

At the end of both induction and withdrawal phases, the expression levels of miR-146a and miR-155 in ADI and ADW rats were significantly upregulated as compared to the respective control group (CI and CW). In addition, ADT rats expressed significantly higher levels of miR-146a and miR-155 than that of the respective control rats (CT) at the end of therapeutic phase. Regarding the effects of MSCs and acitretin, ADTS, ADTA and ADTSA groups showed significantly compensatory effect in comparison with the respective control groups (CT and ADT), and ADTSA group recorded the best one (*P* ≤ 0.05) (Fig. 3A and B).

Regarding the signal molecules that related to NF-kB signaling pathway, ADI and ADW were significantly upregulated for TNFAIP1, TGF-βRII, IL-1β expression levels as compared to the respective control groups (CI and CW) at the end of both induction and withdrawal phases. Similarly, at the end of therapeutic phase, untreated group (ADT) expressed significant higher levels of its genes than the respective control group (CT) which has been compensated after injections with MSCs and acitretin and ADTSA showed the best compensatory one (*P* ≤ 0.05) (Fig. 3C,D and E).

On the other side, no significant change of IL-4 expression was occurred in AD group (ADI and ADW) as compared to the respective control groups (CI and CW) at the end of both induction and withdrawal phases. In addition, untreated group (ADT) expressed higher levels of its genes than the respective control group (CT) at the end of the therapeutic phase. Regarding the role of MSCs and acitretin, ADTS, ADTA and ADTSA groups were significantly upregulated as compared to the respective controls (CT and ADT), and ADTS group recorded the best one (*P* ≤ 0.05) (Fig. 3F).

## Comet assay

### Tail length, tail DNA percentage and tail moment

**Figure 4:**
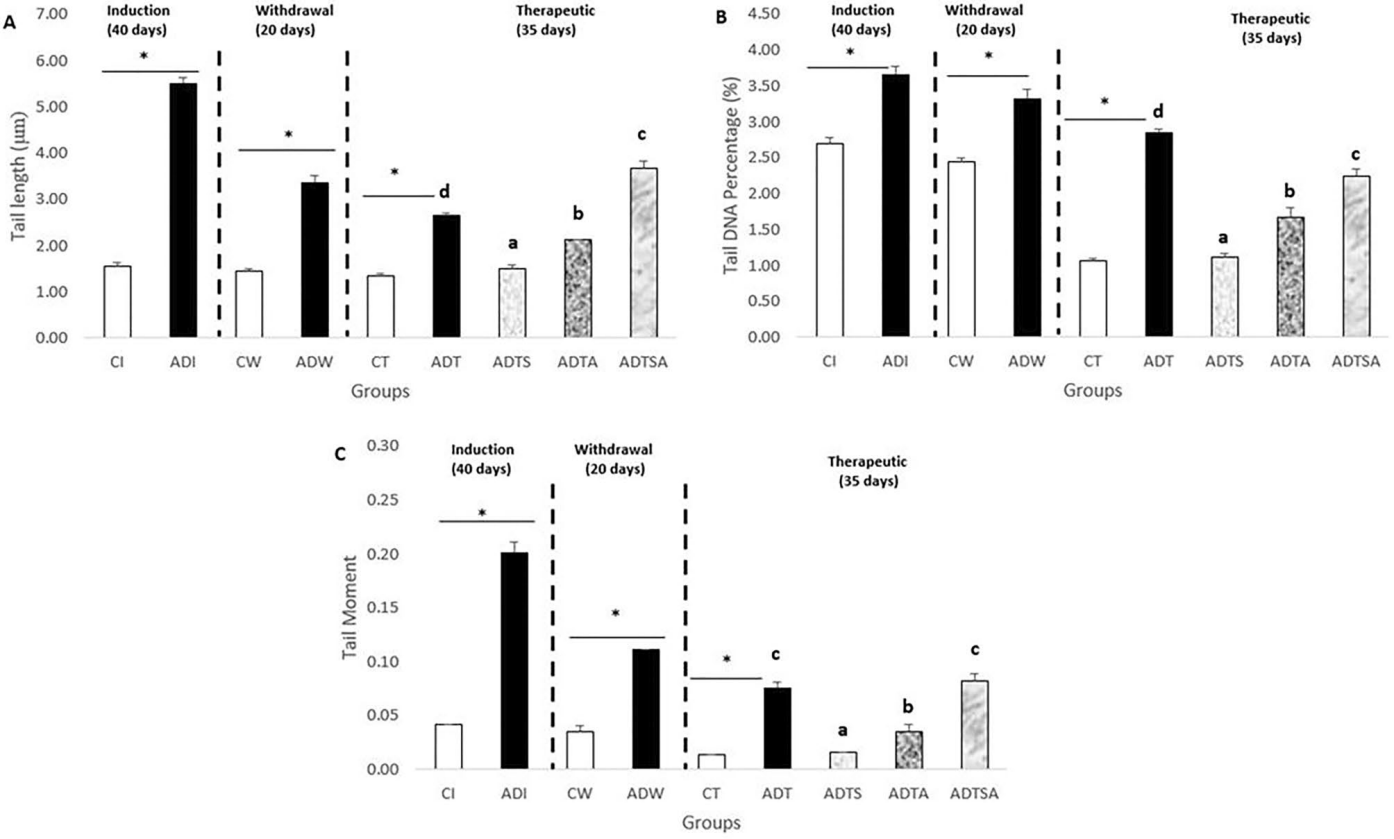
Variations in whole blood (**A**) tail length, (**B**) tail DNA percentage and (**C**) tail moment in AD-like rat model throughout the whole experimental period, the expression of values is using mean ± SD; *P* ≤ 0.05 significant difference compared to both control and treated groups. *: *P* ≤ 0.05 significant difference compared to both control and treated groups. One-way ANOVA followed by Duncan a, b post hoc Test was done, and statistical significance is expressed by different letters. Groups having different small letters are significantly different at *P* ≤ 0.05, while groups having the same small letters are non-significantly different *P* > 0.05.

At the end of both induction and withdrawal phases, ADI and ADW rats displayed significantly longer tail length, higher tail DNA percentage and higher tail moment than that of control rats (CI and CW). At the therapeutic phase, ADT rats showed significantly longer tail length, higher percentage of tail DNA and higher tail moment than that of control rats (CT). ADTA and ADTSA groups exhibited longer significant tail length, higher tail DNA percentage and higher tail moment compared to control rats (CT). ADTS rats showed the significant shortest tail length and the lowest tail DNA percentage and tail moment compared to ADTA and ADTSA rats and nearly reached equal tail length, tail DNA percentage and tail moment in comparison with control rats (CT). ADTSA had the longest tail length and the highest tail moment among all groups of the therapeutic phase (*P* ≤ 0.05) (Fig. 4A,B and C).

### Homing

**Figure 5:**
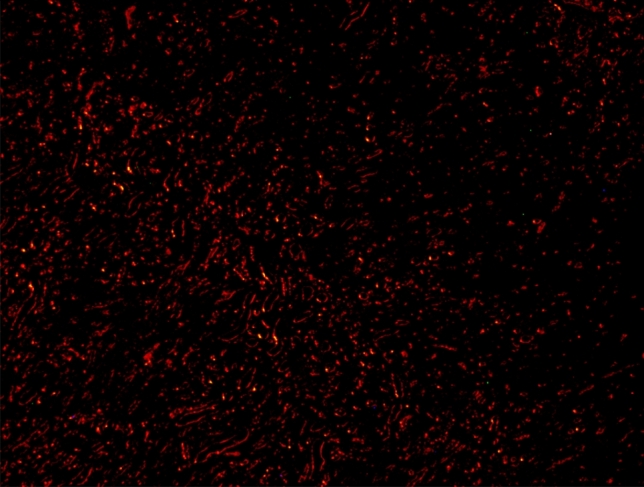
Homing of fluorescent ADMSCs in the brain of AD-like rat model after 2 days ADMSCs injection.

For further confirmation, In vivo fluorescence measurements indicated that the red PKH26 fluorescently labeled ADMSCs which were injected through tail vein dislodged successfully to AD brain after 2 days from injection (Fig. 5).

### Histopathology examination

**Figure 6:**
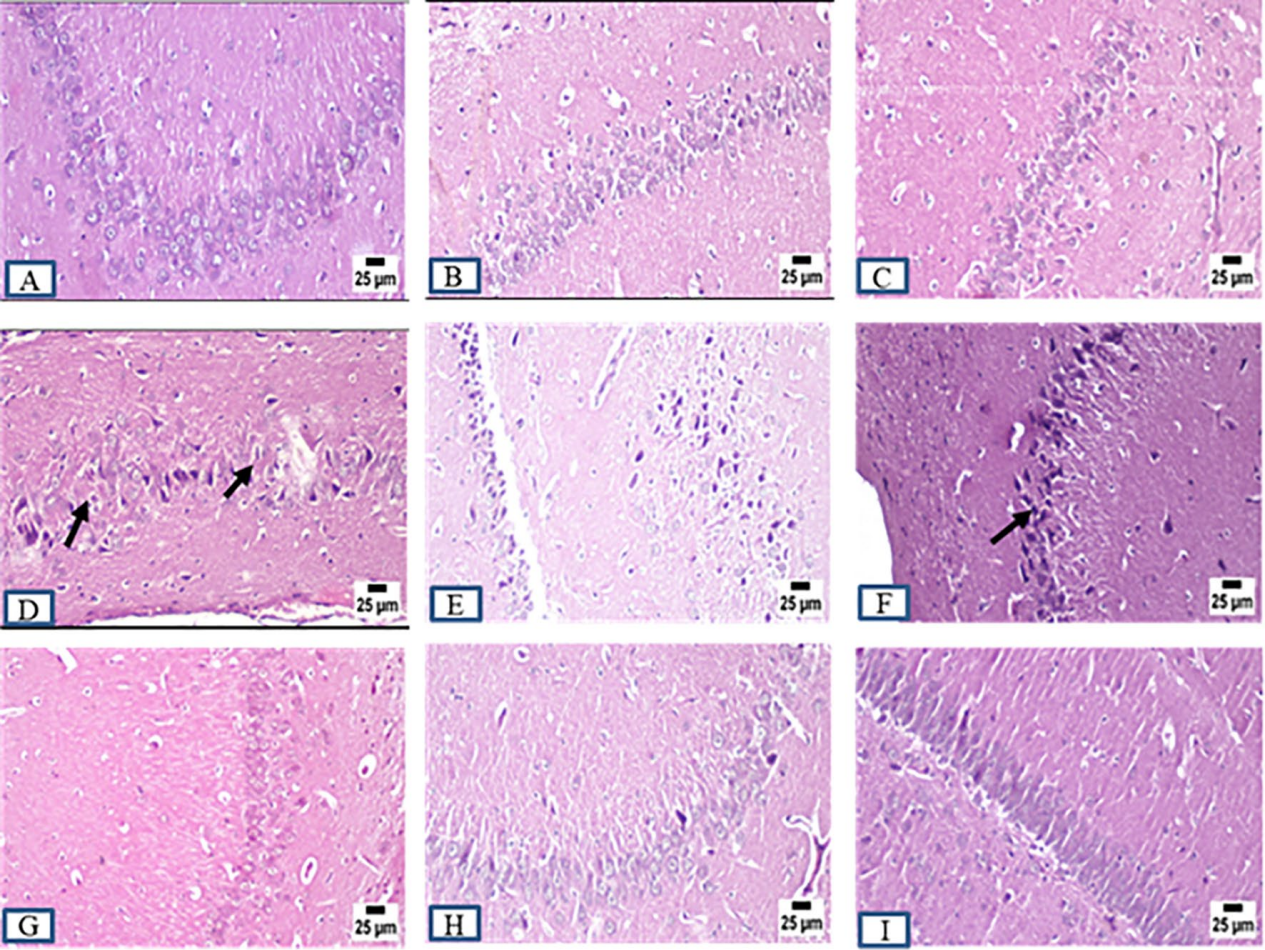
Sections were taken from the brains of (**A**) CI, (**B**) CW, (**C**) CT, (**D**) ADI, (**E**) ADW, (**F**) ADT, (**G**) ADTS, (**H**) ADTA (**I**) ADTSA rat groups throughout the whole experimental period in hippocampus.

**Figure 7:**
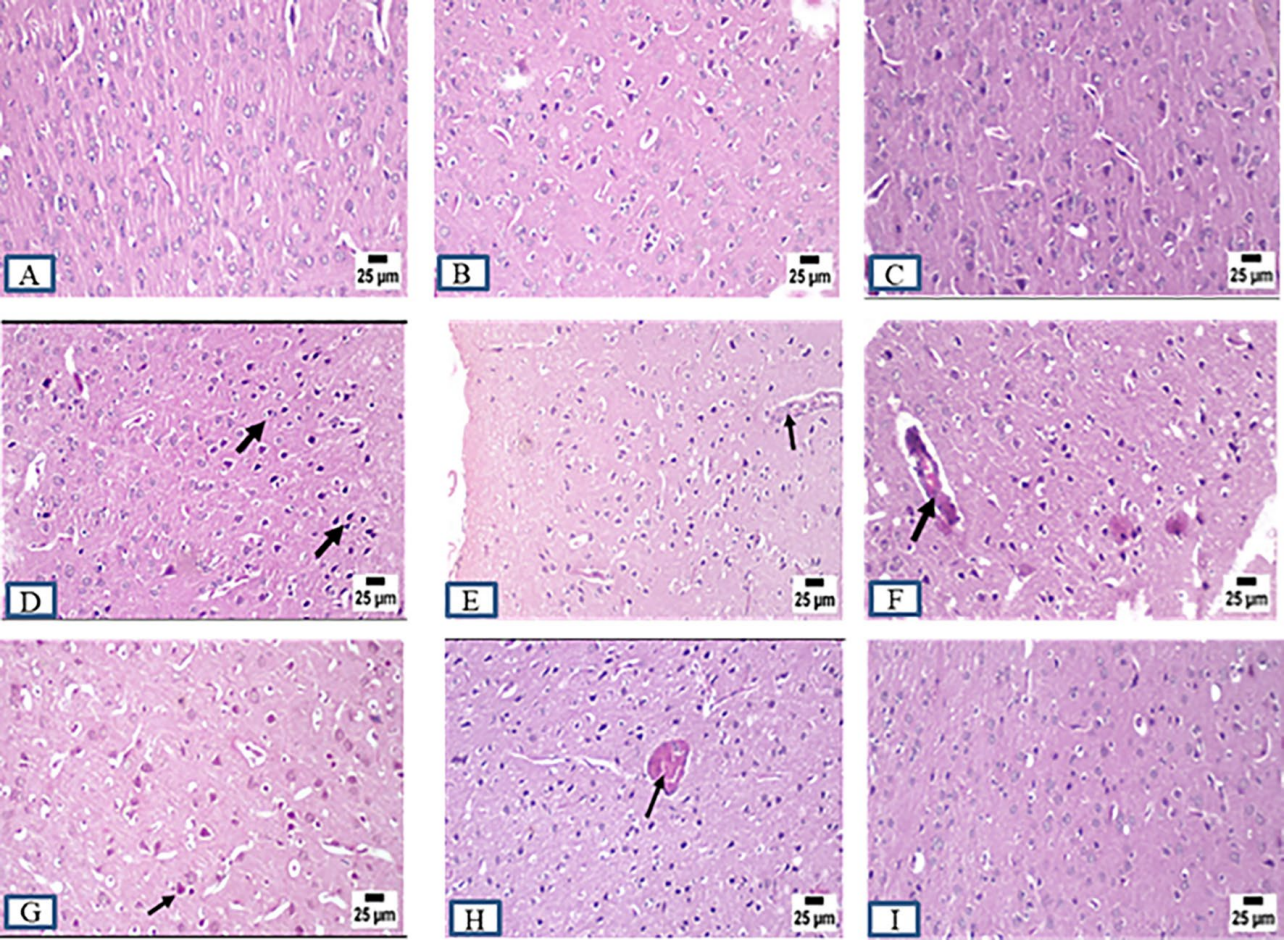
Sections were taken from the brains of (**A**) CI, (**B**) CW, (**C**) CT, (**D**) ADI, (**E**) ADW, (**F**) ADT, (**G**) ADTS, (**H**) ADTA (**I**) ADTSA rat groups throughout the whole experimental period in cerebral cortex.

**Figure 8:**
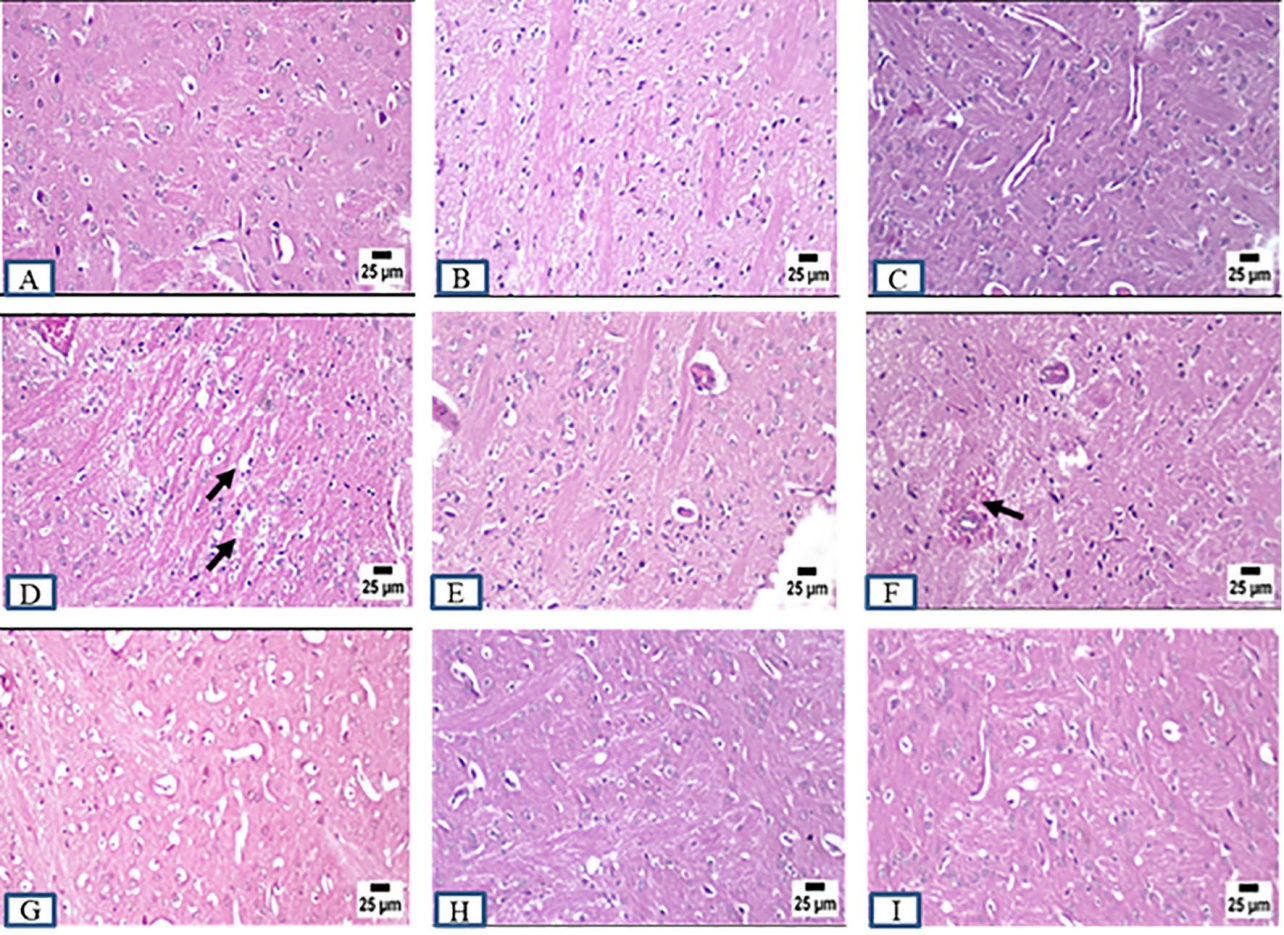
Sections were taken from the brains of (**A**) CI, (**B**) CW, (**C**) CT, (**D**) ADI, (**E**) ADW, (**F**) ADT, (**G**) ADTS, (**H**) ADTA (**I**) ADTSA rat groups throughout the whole experimental period in striatum.

**Figure 9:**
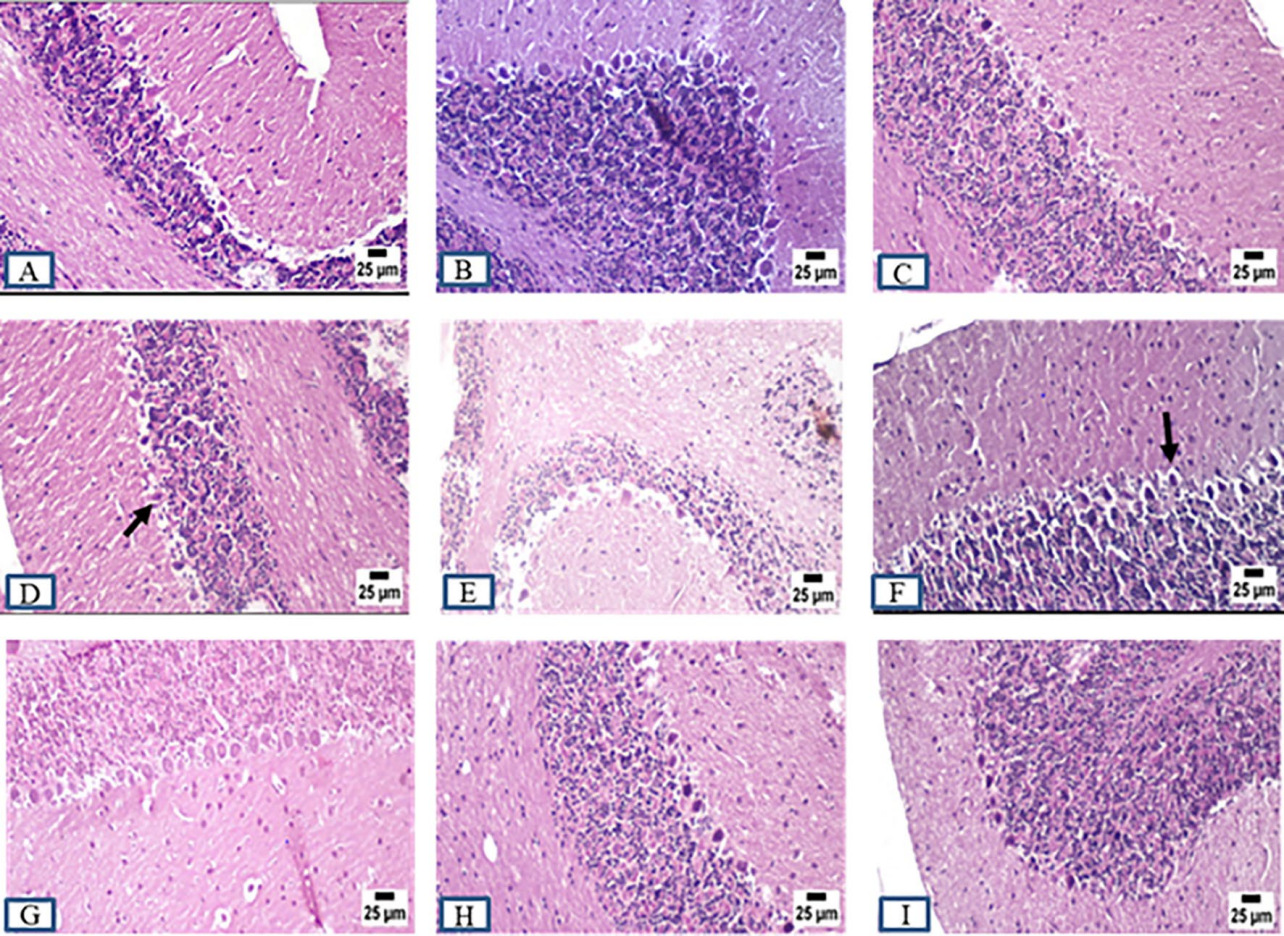
Sections were taken from the brains of (**A**) CI, (**B**) CW, (**C**) CT, (**D**) ADI, (**E**) ADW, (**F**) ADT, (**G**) ADTS, (H) ADTA (I) ADTSA rat groups throughout the whole experimental period in cerebellum.

ADI and ADW rats showed remarkable changes at the histopathological level compared to control rats (CI and CW) at different brain regions including cerebral cortex, striatum, hippocampus, and cerebellum at the end of both induction and withdrawal phases. Control rats exhibited normal histopathological features at different brain regions at the end of all experimental phases. AD rats displayed marked histopathological changes including different degrees in neuronal degeneration in the cerebral cortex and hippocampus at the end of all experimental phases. Marked vacuolation (a cell that has been shaped into or contains one or more vacuoles or tiny, membrane-bound cavities) and gliosis (CNS reaction to brain or spinal cord injury) were observed in the striatum at the end of all experimental phases. Cerebellum Purkinje cell necrosis was clearly observed at the end of all experimental phases accompanied by more acidophilic cytoplasm with pyknotic or completely absent nuclei. Concerning the role of MSCs and acitretin, ADTS group showed improvement with greating power in degeneration with minimal degenerating cells and mild perivascular edema within the cerebral cortex and the striatum respectively while the hippocampus and cerebellum were apparently normal. ADTA group showed normal histopathological features at different brain regions except for a perivascular hemorrhage in the cerebral cortex while ADTSA group showed the best one with an apparently normal brain (Figs. 6,7, 8, and 9).

## Discussion

Alzheimer’s disease is a progressive neurodegenerative disease characterized by symptoms such as memory loss and changes in cognitive function^20^. Amyloid beta plaques (implicated in Alzheimer's disease exists in numerous molecular forms that accumulate between neurons) and neurofibrillary tangles are considered the most common hallmarks of AD^21^. Only the Acetylcholinesterase enzyme inhibitors (AChE) and N-methyl d-aspartate (NMDA) antagonists are the FDA approved drugs for AD and they only improve the symptoms but do not prevent the progression of AD^22,23^.

In the present study, AD-like rat model was established by oral successive doses of AlCl_3_ for 40 days followed by drug withdrawal for 20 days and treatment by MSCs and/or acitretin for 35 days. The purpose of this study was to expose the therapeutic effect of mesenchymal stem cells (MSCs) and/or acitretin on NF-kB inflammatory signaling pathway-associated genes (TNFAIP1, TGF-βRII, IL-1β and IL-4) and its regulatory miRNAs namely, miR-146a and miR-155 in AD-like rat model.

As AlCl_3_ model of Alzheimer’s disease is still a questionable by several scientists as real AD model, and AD is well-known as a progressive neurodegenerative disease, the present study was interested to prove this concept by suggesting a withdrawal phase before the onset of therapeutic phase. AlCl_3_ intoxication should be accompanied by a reversible effect during the withdrawal phase, but this was not the case in the current model as AlCl_3_ withdrawal was accompanied by a progressive neurodegeneration which is a major feature of AD, a point which needs more clarification.

The induction of AD in rats was obviously successful and the incidence of dementia was demonstrated through behavioral changes as recently reported by ^24^ and changes in levels of lipid peroxidation and antioxidant defense system. Regarding the oxidative stress markers, pro-oxidative marker (MDA) showed a significant increment in AD group as compared to the respective controls at the end of all experimental phases. In opposition, anti-oxidative defense system which represented by (GSH and SOD) were significantly inhibited in ADI as compared to respective control (CI) at the end of the induction phase. Concerning the role of stem cells and acitretin, MDA was significantly decreased in all therapeutic groups (ADTS, ADTA, ADTSA) as compared to the respective control (ADT). On the other hand, anti-oxidative defense system (GSH and SOD) was significantly stimulated in ADTS and ADTSA as compared to ADT. Besides there was a continuous rise of brain β- amyloid level through induction, withdrawal, and therapeutic phases in AD rats, which has not been unfortunately compensated by the different therapeutic regimens, a point which needs further investigation. Oppositely, a high significant serum β- amyloid level in AD rats has been observed in comparison with the respective control during the whole experimental phases which has been compensated by different therapeutic regimens. In addition, histopathological findings proved the ability of different therapeutic regimens bring to almost their normal appearance.

At the molecular level, there were significant changes in the expression levels of various miRNAs including miR-146a and miR-155, associated genes including (TNFAIP1 and TGF-βRII) and cytokines inflammatory genes including IL-1β and IL-4 related to NF-kB pathway. The Comet assay represented another proof for AD development through the variation in tail length, tail DNA percentage and tail moment indicating DNA fragmentation. Eventually, homing illustrated the success of MSCs to pass the brain barriers and be implemented in the brain tissues (the site of injury).

In the following paragraphs illustrated the full description of the present findings starting with hallmarks of AD including β-amyloid. The present data showed a significant increase in serum and brain Aβ levels of AD rats versus control rats during all three experimental phases which run in agreement with many previous studies that have been reported by^25–27^. The efficacy of different treatments was not remarkable as there were no significant changes in brain Aβ levels after administration for AD rats. In contrary, different treatments were clearly effective in restoring serum Aβ levels nearly reaching the control values. Among therapeutic groups, ADTS showed the best one. The best explanation that might justify these non-significant changes in brain Aβ is that extended length of the therapeutic phase is needed to regenerate more neurons accompanied by reduced β-amyloid deposition.

Oxidative stress is considered as one of the main causes for the onset and progression of AD^28^. A significant increase in MDA levels and a decrease in GSH levels of AD rats compared to control rats were observed in whole experimental phases, while SOD levels in AD rats were decreased significantly in comparison with control rats only at the end of the induction phase which might be attributed to AlCl_3_ accumulation in blood. These findings run in agreement with many previous studies that have been conducted by^3,28–30^. After the administration of various treatments, MDA was significantly declined in all therapeutic groups (ADTS, ADTA, ADTSA) as compared to the respective control (ADT). In contrast, GSH and SOD were significantly induced in ADTS and ADTSA as compared to ADT which are confirmed by previous reports of^3,28–30^.

In the present study, molecular measurements were performed by measuring the gene expression levels of two miRNAs (miR-146a and miR-155), related genes (TNFAIP1, TFG-βRII) and inflammatory cytokines (IL-1β and IL-4) associated to NF-kB pathway. A significant upregulation was detected in the expression levels of both genes (miRNAs, TNFAIP1, TFG-βRII and IL-1β) except for IL-4 during induction and withdrawal phases which is an anti-inflammatory cytokine associated with roles in the maintenance of learning and memory providing evidence for AD neurodegeneration. During the therapeutic phase, the expression levels of miRNAs and related genes (TNFAIP1, TFG-βRII and IL-1β) were significantly downregulated except for IL-4 after the administration of various treatments demonstrating their high efficacy in minimizing the effects of AD. Among the therapeutic regiments, the combination group of MSCs and acitretin showed the best compensatory effect. The present study agrees with many previous studies that are conducted by^3,31–39^.

Comet assay is commonly used to detect genotoxicity, and that is general indicators for DNA fragmentation accompanying Al intoxication. A significant increase in the parameters of comet assay namely tail length, tail DNA percentage and tail moment was observed in AD rats in comparison with control rats demonstrating the neurotoxic effect of Al during the three experimental phases which agrees with previous studies that have been done by^40,41^. After the administration of various treatments, the DNA fragmentation level was compensated and ADTS group recorded the best efficacy.

In vivo fluorescent measurements confirmed the success of red PKH26 fluorescently labeled ADMSCs migration to the damaged brain tissue (the site of injury) and that in agreement with^24,42^. Regarding the histopathological findings, there were remarkable changes in different brain areas at the histopathological level including degenerated neurons specially within the hippocampus and cerebral cortex. Vacuolation and gliosis with observed thickening of blood vessels wall at the striatum and Purkinje cell necrosis at the cerebellum in AD rats were exhibited as compared to control. These findings expose the neurodegenerative effect of AlCl_3_ in AD rats at the histopathological level and which agrees with previous findings^28,43,44^. Administration of various treatments almost retrieved most of the brain regions to their normal state except the cerebral cortex and striatum in ADTS and cerebral cortex only in ADTA. The combined treatment of MSCs and acitretin showed the best results.

According to other researchers, intranasal stem cells control and treat brain tumors^45^, protect against the neurologic side effects of radiation^46^, and treat chemotherapy-induced peripheral neuropathy^47^. Mesenchymal stem cells given orally have also been shown to transfer their mitochondria to neural stem cells to stimulate the production of brain cells' energy^48^.

By eliminating the need for intravenous delivery, which disperses cells throughout the body and causes unintended systemic exposure, as well as the need for invasive neurosurgical implantation of cells, this intranasal delivery, directing, and treatment technology can make stem cell treatments for brain disorders feasible. This method of treatment may pave the way for the creation of stem cell, immune cell, microglia, macrophage, and genetically engineered cell therapies for Parkinson's, PSP, and Huntington's^49^, Alzheimer's^50^, MS, epilepsy, stroke, neonatal ischemia, brain tumours, traumatic brain injury (TBI), and even LSDs like Niemann-Pick type C disease^51^. According to a recent report by Danielyan et al.^53^, choosing stem cells with superior cell motility and enhanced migratory potential can increase the therapeutic benefit obtained after intranasal stem cell therapy.

## Conclusion

**Figure 10:**
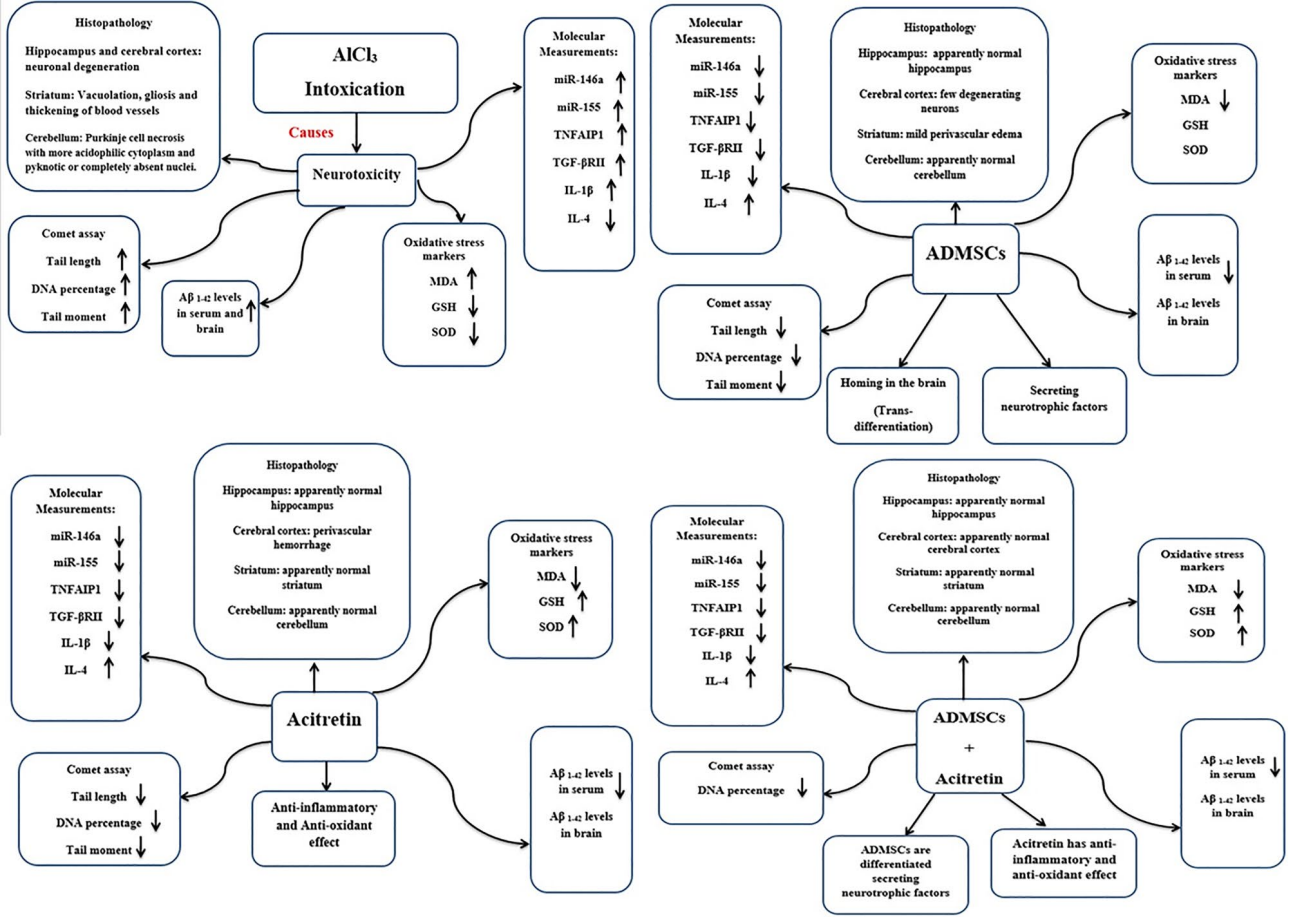
Schematic representation of Alzheimer's disease signs after AlCl_3_ intoxication, compensatory effect of an administration of ADMSCs, compensatory effect of an administration of acitretin, and compensatory effect of an administration of ADMSCs and/or acitretin in AD-like rat model.

As summarized in (Fig. 10), the induction of AD in rats was obviously successful and the incidence of dementia was demonstrated through changes in levels of lipid peroxidation and antioxidant defense system. Overall, the present study demonstrated the therapeutic potential of the utilized treatments for restoring the normal state of AD-like rat model at the histopathological, physiological, and molecular levels after the appearance of memory deficits and signs of dementia. The combined treatment of MSCs and acitretin showed the best results for compensating the neurodegeneration that caused by AlCl_3_. These findings can give hope for the development of therapy to treat AD and stop its progression not only to improve the symptoms. The present study also confirmed the eligibility of using miR-146a and miR-155 as additional biomarkers for diagnosis and treatment of AD.

### Limitation of the study

There was a continuous rise of brain β- amyloid level through induction, withdrawal, and therapeutic phases in AD-like rat model, which has not been unfortunately compensated by the different therapeutic regimens under the experimental conditions, a point which needs further investigation. Despite the fact that different treatments were clearly effective in restoring serum Aβ levels nearly reaching the control values, extending the length of the therapeutic phase seems necessary to regenerate more neurons and restore normal or almost levels of β-amyloid deposition in brain tissues.

### Future respective

The promising changes in brain levels of miR-146a and miR-155 documented in the present study during the three experimental phases need some support by evaluating the circulating levels as far as they are used as eligible biomarker for Alzheimer’s disease and is a point of future respective.

## Methods

### Animals

Fourty-five male albino rats (Rattus norvegicus) with approximate weight 150–200 g were purchased from a commercial supplier (National Research Center, Dokki, Egypt). The rats were housed in cages in specific pathogen-free conditions and a 12-h light/12-h dark cycle at a constant temperature of (~ 25 °C) with free access to normal chow diet and water. The rats were supposed to one week before performing the experiments for adapting to the environment.

### Animal rights

Experimental protocols and procedures used in this study were approved by the Cairo University Institutional Animal Care and Use Committee (CU-IACUC) (Egypt), (approval no. CU/I/F/51/20) in accordance with the international guidelines for care and use of laboratory animals.

### Experimental design and analysis

The rats were randomly divided into negative control groups (n = 15) and Alzheimer’s disease groups (n = 30). The experimental periods were divided into induction phase (40 days), withdrawal phase (20 days) and therapeutic phase (35 days).

At the induction phase, rats were classified into control group (CI) given orally 1 ml of distilled water daily and Alzheimer’s disease group (ADI) given orally 100 mg/kg/BW/daily AlCl_3_.5 H_2_O^54^. At the withdrawal phase, no treatment was performed. At the therapeutic phase, rats were categorized into control group (CT) given orally 1% DMSO diluted in saline daily, AD group (ADT) left untreated, AD group injected by stem cells only (ADTS) intravenously injected in tail vein by a single dose of red PKH26 fluorescently-labeled ADMSCs (1.0 × 10^6^ cells/0.5 ml serum supplemented DMEM/rat)^55^, AD group injected by acitretin only (ADTA) given an oral dose of acitretin (2.5 mg/kg/day) dissolved in 1% DMSO diluted in saline^56^ and AD group injected by both stem cells and acitretin (ADTSA) given a combined treatment with ADMSCs and acitretin daily. At the end of each phase, the rats were euthanized by using sodium pentobarbital (50 mg/kg) to obtain blood and tissues for further studies.

### Experimental procedures

#### Blood and tissue collection

At the end of each phase, half of the rats from each group were anesthetized with 50 mg sodium pentobarbital per kg then euthanized by cervical dislocation for blood collection from the retro-orbital plexus^57^. Serum was separated via coagulation and centrifugation at 3000 rpm for 10 min for further experiments. The brain was excised, washed with saline, dried on filter paper, and weighed. The brain was dissected into two halves; one half was stored in 10% formalin for histopathological tests and homing of various brain area. The other half was homogenized using 10% (w/v) phosphate buffer saline (PBS) and stored at − 80 °C for further assays.

#### Enzyme-linked immunosorbent assay (ELISA)

The present study evaluated the serum and brain Aβ1-42 levels in rat groups at the end for each experimental phases using a sandwich ELISA according to manufacturer’s instructions (Nova, Beijing, China).

#### Oxidative stress biomarkers

The present study measured the activity of serum MDA, GSH, and SOD by using spectrophotometer according to manufacturer’s instructions (Biodiagnostic, Dokki, Giza, Egypt) according to the method of Ohkawa et al.^58^ for lipid peroxidation (MDA), Aykaç et al.^59^ for reduced glutathione (GSH), Kakkar et al.^60^ for superoxide dismutase (SOD), respectively.

### Quantitative real time-PCR (RT-QPCR)

Total RNA was isolated using the RNeasy Mini kit (Qiagen, Germany) according to the manufacturer’s instructions. Then synthesis of cDNA from miRNA was performed using the miScript® II RT Kit (Qiagen, Germany) according to the manufacturer’s instructions. GAPDH was used as a control primer and the targeted genes including miR146a, miR-155, TNFAIP1, TGF-βRII, IL-1β and IL-4. Primers used for RT- qPCR were commercially synthesized from (Macrogen, Seoul, South Korea), and their sequences are listed in Table 1. RT-qPCR was performed in applied Biosystems Step One Plus (Thermo Fisher Scientific, Inc., Waltham, MA, USA) using HERAPLUS SYBR® Green qPCR Kit (Willowfort, UK), and each sample was prepared as triplet for each gene. Each sample was initially denatured at 94 °C for 4 min followed by 40 cycles at 94 °C for 1 min, 56 °C for 1 min and 72 °C for 1 min. Relative expression levels of the miRNAs and genes were calculated using the 2^−ΔΔCt^ method^61,62^.

**Table 1:** Primer sequences of genes involved in RT-qPCR.

GAPDH	Forward: 5′-AGTGCCAGCCTCGTCTCATA-3′ Reverse: 5′-GATGGTGATGGGTTTCCCGT-3′^66^
miR-146a	Forward: 5′- TCTGAGAACTGAATTCCATGGGT -3′ Reverse: 5′-TGACGATAGAGCTATCCCAGC -3′
miR-155	Forward: 5′-TGTGATAGGGGTTTTGGCCTC -3′ Reverse: 5′-TGTTAATGCTAACAGGTAGGAGTC -3′
TNFAIP1	Forward: 5′—TACCTCCGAGATGACACCGT-3 ′ Reverse: 5′—CACCAGCCCTTGAATGAGGT -3′
TGF-βRII	Forward: 5'- ATCCTGAGAGGGCGAGGAAT -3' Reverse: 5'- GCTGTTAACCGACTTGGGAAC-3'
IL-1B	Forward: AGGCTGACAGACCCCAAAAG Reverse: CTCCACGGGCAAGACATAGG
IL-4	Forward: GTACCGGGAACGGTATCCAC Reverse: ACATCTCGGTGCATGGAGTC

### Comet assay

The DNA fragmentation in the cells of rat groups during the end of each experimental phase was evaluated using comet assay^63^. The alkaline comet assay was done as described by^64^. The whole blood was dissolved in 0.75% low melting-point agarose and immediately spread onto a glass microscope slide pre-coated with a layer of 1% normal melting-point agarose. The slides were then incubated in ice-cold lysis solution (2.5 M NaCl, 10 mM Tris, 100 mM EDTA, 1% Triton X-100, and 10% DMSO, pH 10.0) at 4 °C for at least 1 h to get rid cellular proteins and membranes. Slides were removed from the lysis solution and placed on a horizontal electrophoresis unit. The unit was filled with fresh buffer (300 mM NaOH, 1 mM EDTA, pH13.0), which covered the slides for 20 min at 4 °C to allow unwinding of DNA and expression of alkali-labile sites. Electrophoresis was performed for 20 min at 25 V (74 V/cm). Slides were then neutralized (0.4 M Tris, pH 7.5), fixed in absolute ethanol for 10 min and then slides were left to dry at room temperature, The neutral comet assay was conducted at pH 8.5, essentially according to the same procedure as the alkaline version, except at lower pH. In the neutral version, electrophoresis was carried out in a buffer consisting of 100 mM Tris and 300 mM sodium acetate at pH 8.5.

The slides were stained with ethidium bromide and analyzed under a fluorescent microscope. One hundred cells (50 cells from each of two replicate slides) per concentration of each test substance were selected and analyzed visually with an optical microscope for tail length and amount of DNA present in the tail. When selecting cells, the areas around air bubbles or at the edges were prevented^65^. The parameters selected for analysis were: (1) the tail length (TL) (the distance between the center of the comet head and the last non-zero pixel of the comet profile), (2) percentage of DNA in tail (% DNA) (the intensity of all tail pixels divided by the total intensity of all pixels in the comet, expressed as percentage, (3) tail moment (TM) (equivalent to the % DNA in the comet tail multiplied by the tail length) and (4) olive tail moment (OTM)—computed as the summation of each tail intensity integral value, multiplied by its relative distance from the center of the head, the point at which the head integral was mirrored, and divided by the total comet intensity.

### Homing

Two rats were selected randomly from stem cell treated group and injected intravenously through tail vein with a red PKH26 fluorescently labeled in a single dose of 1 × 10^6^ ADMSCs. Fluorescence images were picked up after 2 days post-transplantation using fluorescent-inverted microscope for assessing migration of stem cells to injured organ (homing).

### Histopathology of brain tissues

Fixed brain tissues were dehydrated, cleared, and embedded in paraffin. The paraffin blocks were cut using microtome in thin tissue sections (6 µm) and stained by hematoxylin and eosin (H&E) for subsequent histopathological examinations. Unstained brain tissue sections were examined by using fluorescent-inverted microscope for assessing homing.

### Statistical analysis

Values were expressed as (mean ± SD). Statistical analysis was performed using SPSS statistical software package version 25 by one-way analysis of variance (ANOVA) with Duncan post hoc test and *P* ≤ 0.05 was considered statistically significant.

## Data Availability

All data generated or analyzed during this study are included in this published article. The datasets generated and/or analyzed during the current study are available and accession numbers of datasets are XM_039110643.1, NR_106710.1, NM_182950.4, NM_031132.4, NM_031512.2, and NM_201270.1 and NM_017008.4. In addition, the direct web links are NM_182950.4 Rattus norvegicus TNF alpha induced protein 1 (Tnfaip1), mRNA, https://www.ncbi.nlm.nih.gov/nucleotide/119310187, NM_031132.4 Rattus norvegicus transforming growth factor, beta receptor 2 (Tgfbr2), mRNA, https://www.ncbi.nlm.nih.gov/nucleotide/1936500939, XM_039110643.1 PREDICTED: Rattus norvegicus forkhead associated phosphopeptide binding domain 1 (Fhad1), transcript variant X9, mRNA, https://www.ncbi.nlm.nih.gov/nucleotide/1958778177, NM_031512.2 Rattus norvegicus interleukin 1 beta (Il1b), mRNA, https://www.ncbi.nlm.nih.gov/nucleotide/158186735, NM_201270.1 Rattus norvegicus interleukin 4 (Il4), mRNA, https://www.ncbi.nlm.nih.gov/nucleotide/42627876, NR_106710.1 PREDICTED: Rattus norvegicus microRNA 155 (Mir155), microRNA, https://www.ncbi.nlm.nih.gov/nuccore/NR_106710.1, NM_017008.4 Rattus norvegicus glyceraldehyde-3-phosphate dehydrogenase (Gapdh), mRNA, https://www.ncbi.nlm.nih.gov/nucleotide/402691727.
